# Ecological predictors of academic satisfaction in senior secondary school students in Hong Kong: The mediating role of academic confidence

**DOI:** 10.3389/fpsyg.2022.1041873

**Published:** 2022-11-29

**Authors:** Diya Dou, Daniel T. L. Shek, Tingyin Wong

**Affiliations:** Department of Applied Social Sciences, The Hong Kong Polytechnic University, Hunghom, Hong Kong SAR, China

**Keywords:** NSS, Hong Kong, senior secondary students, academic satisfaction, positive youth development, academic confidence, academic stress, school support

## Abstract

**Introduction:**

Although the secondary school curriculum reform has taken place for more than 1 decade in Hong Kong, very few studies have examined senior secondary school students’ academic satisfaction and its predictors at the individual and school levels. The present study examined the influence of academic stress, school support, positive youth development (PYD) attributes on academic satisfaction via the mediation of academic confidence among senior secondary school students using three-wave longitudinal data.

**Methods:**

This study was derived from a 6-year longitudinal project examining youth development among Hong Kong adolescents. Only three waves of data collected from 2,023 students, including 959 boys (47.4%) and 1,040 girls (51.4%), from grade 10 to 12 (i.e., Waves 4–6), were used in the present study focusing on senior high school years. Students responded to a questionnaire concerning different aspects of their development, including their perceptions of school support, PYD attributes, academic stress, academic confidence, and satisfaction with the NSS curriculum. We conducted structural equation modeling (SEM) to test the hypothesized model.

**Results:**

Results showed that while Wave 4 academic stress negatively predicted academic satisfaction at Wave 6, Wave 4 positive youth development attributes and school support had positive associations with Wave 6 academic satisfaction; Wave 5 academic confidence also served as a mediator in these relationships, except for the relationship between school support and academic satisfaction.

**Discussion:**

The theoretical, practical, and policy implications of the findings are discussed. The present study generally supports previous findings on the relationships between academic stress, school support, PYD attributes, academic confidence, and academic satisfaction. The findings emphasize the prominence of PYD attributes, school support, and confidence in enhancing students’ academic satisfaction.

## Introduction

Students’ academic satisfaction refers to their perceptions of and satisfaction with academic experiences (Jadidian and Duffy, 2011). Marchiondo defined it as “the attraction or positive feelings that a student associates with the college or program” (Marchiondo et al., 2010, p. 610). It is also an essential indicator of students’ well-being and education quality (Simonsen and Rundmo, 2020). Scholars have found that students with high levels of academic satisfaction tend to be engaged actively in learning activities, put much effort into fulfilling tasks, and perform better (Singh et al., 2002). Rowe et al. (2007) found that positive perceptions of learning experiences in schools also could strengthen students’ health and well-being. However, students who do not like or feel disconnected from schools tend to experience lower academic performance, a higher risk of dropping out, and diminished well-being (Shochet et al., 2006; Archambault et al., 2009).

Various factors at the individual and environmental levels influence students’ academic satisfaction. According to ecological theory of Bronfenbrenner (1979), adolescents’ development is embedded in a multitude of social relationships at different levels, including individual, family, school, community, and societal. Researchers have argued that environmental and personal factors can be protective or function as risk factors in shaping adolescent academic satisfaction and well-being (Benson et al., 2011). At the environmental level, supportive and healthy school environments can protect and promote young people’s health, while a less-supportive school environment, including from teachers, leads to negative outcomes (Currie et al., 2012). At the individual level, adolescents’ positive youth development (PYD) attributes and academic confidence contribute to students’ development and well-being (Benson et al., 2007). Broadly speaking, PYD attributes refer to external and internal developmental assets contributing to thriving in adolescents (Shek et al., 2019).

Despite extant studies identifying predictors of academic satisfaction, this issue should be understood using an ecological framework with reference to specific contexts. Considering the lack of related studies conducted with Hong Kong students adjusting to education reform measures, the present study examined ecological factors’ influence at the individual and school levels on students’ academic confidence and satisfaction with the curriculum, using longitudinal data collected during senior secondary school years.

### Academic satisfaction

Satisfaction reflects one’s perceptions of and liking for specific aspects of an environment. For students, educational environment is prominent for their knowledge increasing, cognitive development, intra- and inter-personal development, and school performance (Flores, 2007). Academic satisfaction represents students’ intrinsic gratification through academic pursuits (Ryan, 2001). It is closely linked to learning outcomes, life satisfaction, and social development (Flores, 2007). It is an important indicator of students’ well-being and a marker of academic success (Simonsen and Rundmo, 2020). Sanders and Burton (1996) found that college students’ academic satisfaction is the most significant predictor of their personal satisfaction.

Scholars have focused on different aspects of academic satisfaction (Lent et al., 2016). For example, Lodi et al. (2017) developed a scale with five domains to assess college students’ satisfaction, including appropriateness of the student’s choice, quality of the University services, relationships with his/her colleagues, and quality of his/her study habits and usefulness for his/her future career. Nauta (2007) mainly focused on students’ satisfaction with their major or study field. Many studies evaluating educational effectiveness focusing on students’ perceptions of courses and classes (Byrne and Flood, 2003; Jadidian and Duffy, 2011), particularly when significant changes occurred in educational settings (e.g., flipped classroom), pedagogies (e.g., experiential learning), and the external environment (e.g., the outbreak of COVID-19, Montero et al., 2022; Polat and Karabatak, 2022). Given that Hong Kong implemented an educational reform introducing significant curricular changes, the present study focuses on students’ perceptions of the curriculum, which reflects the attraction and effectiveness of the curriculum (Marchiondo et al., 2010; Jadidian and Duffy, 2011).

### Ecological factors and academic satisfaction

Ecological theory of Bronfenbrenner (1979) provides a framework for understanding how adolescents’ development is influenced by nested ecological systems at different levels. Scholars have identified some factors that influence students’ academic satisfaction, including academic stress, PYD attributes, and academic confidence at the individual level, as well as school support at the environmental level. Among these factors, academic stress is a risk factor that can weaken students’ academic satisfaction, while PYD attributes, academic confidence, and school support are viewed as protective factors that promote students’ development and satisfaction with their school and curriculum.

### Academic stress

According to Putwain (2007), academic stress refers to students’ subjective experience of stress under specific academic-related stimuli or stressors. The sources of perceived academic stress include heavy workload, tight schedules, high demands, and exams (Sarid et al., 2004). PISA 2015 identified “schoolwork-related anxiety” as one of the indicators for measuring negative aspects of adolescents’ well-being. In Organization for Economic Cooperation and Development (OECD) countries, more than 50% of students frequently experience schoolwork-related anxiety, e.g., feeling very anxious about upcoming tests even after thorough preparation. Schoolwork-related anxiety also was found to be associated adversely with academic performance and life satisfaction (OECD, 2017). Besides, Lent et al. (2017) found that academic satisfaction and academic stress covaried significantly.

### School support

School support is the primary environmental factor protecting students against academic stress and promoting positive adjustment to school reform (Torsheim and Wold, 2001). The PISA 2015 report on students’ well-being also viewed students’ sense of belonging at school and their relations with teachers as important areas of students’ lives (OECD, 2017). Remarkably, the report stressed that the quality of relationships between teachers and students could affect students’ socioemotional development, as well as their connection with their schools. Other empirical research also has demonstrated the importance of school support in students’ school satisfaction, academic success, and positive school behaviors. Danielsen et al. (2009) found that teacher support was correlated significantly with students’ school satisfaction. Plenty et al. (2014) concluded that teacher support correlated with students’ positive conduct behaviors, particularly among girls, while support from classmates reduced students’ negative emotional symptoms.

### PYD attributes

As for ecological factors at the individual level, existing studies found that some positive qualities––e.g., self-efficacy, emotional competence, grit, and optimism––had a positive relationship with academic satisfaction. Most existing studies have focused on one or two positive qualities and lack a systematic perspective on students’ positive attributes. Given the recent development in adolescent research comprising a paradigm shift from the problem-based to the strength-based model, the PYD approach has become mainstream in understanding adolescent development. By highlighting resources and strengths’ importance, this approach views adolescents as active agents with the potential to overcome difficulties, experience growth, and thrive (Shek and Ma, 2010; Damon, 2016). The PYD model supports PYD attributes’ protective effect in buffering against stress and facilitating adolescents’ well-being (Gavin et al., 2010; Ma, 2020). However, empirical studies on PYD qualities’ effect on academic stress and satisfaction remain scarce in the literature (Shek and Wu, 2016).

Furthermore, extant studies have examined various positive schoolwork-related attributes of students that had a positive relationship with their academic performance and satisfaction while reducing academic stress. First, scholars investigated students’ self-efficacy beliefs. The self-efficacy attribute is included in the PYD attributes and represents one’s ability to achieve life goals through personal effort (Shek et al., 2019). The self-efficacy beliefs concept originated from Bandura’s social cognitive theory and argued that people’s beliefs about their capabilities affect their behaviors (Bandura, 1977). Education scholars have investigated how students’ self-perceptions influence their academic lives (Pajares, 2007), finding that students’ self-efficacy beliefs could predict positive academic performance (Usher and Pajares, 2008). Second, students with high self-efficacy tend to experience more satisfaction than students with low self-efficacy. Literature found that students’ self-efficacy beliefs could influence their well-being and satisfaction with a course positively (Pajares and Schunk, 2001; DeWitz and Walsh, 2002). Third, scholars have investigated how students’ positive attributes, academic performance, stress, and satisfaction are interrelated. Liu and Lu (2011) found that students with a higher resilience level could surmount the adverse effects of stress on their academic achievement. A recent study of Shek and Chai (2020) indicated that PYD qualities predicted academic satisfaction over time.

### Academic confidence

Another important factor at the individual level is academic confidence. Sander and Sanders (2003) defined academic confidence as students’ beliefs about their capacities to plan and perform behaviors to achieve academic success. According to the self-determination theory (SDT; Deci and Ryan, 2008), academic confidence shows students’ belief in their academic competence and motivation to pursue their academic goals. Suldo et al. (2008) and Wong and Siu (2017) found that students with strong academic confidence reported higher school satisfaction. Chemers et al. (2001) found that students with low academic confidence levels tended to lower their expectations of academic success and achieve less satisfactory results. In the Five Cs model of Richard Lerner, confidence is an important factor contributing to student thriving (Lerner et al., 2005; Shek et al., 2019).

The SDT also provides a theoretical background for the mediation role of self-confidence in the relationship between environmental factors’ effects and students’ academic well-being. Ecological factors may shape their feelings of academic competence, which, in turn, influence their motivation and academic well-being (Deci and Ryan, 2008). For example, when students perceive heavy academic stress, they often experience less control over their studies and decreased confidence. Protective factors, such as school support and individual positive attributes, serve as resources supporting students to manage stress, gain internal locus of control, and strengthen their perceptions of competence. Fariborz et al. (2019) found that self-efficacy played a mediating role in the relationship between academic stress, stress response, and academic burnout. Mampane (2014) and Chapin (2015) found that self-confidence and access to social support enabled students to fight against academic challenges and be successful in school. Zorkina and Nalbone (2003) found that teachers’ instructions influenced students’ confidence levels, which affected their subsequent test performance. In the present study, we analyzed the mediating role of academic confidence in the relationship between students’ academic stress, perceived school support, PYD attributes, and academic satisfaction when facing academic challenges and adjusting to the NSS curriculum. This is consistent with the theoretical prediction of the PYD approach.

### The context of Hong Kong

Hong Kong instituted the New Senior Secondary (NSS) curriculum in 2009 to promote students’ lifelong learning abilities and change Hong Kong’s examination-oriented culture (Fung and Yip, 2010). The NSS curriculum aims to strengthen students’ generic skills and cultivate positive values and attitudes to prepare them for whole-person development. These aims were incorporated into the teaching and learning of each subject by integrating applied learning and theoretical knowledge (Hui et al., 2020). Students also can strengthen these skills and values through the Citizenship and Social Development core subject, and then implement their skills in Applied Learning elective courses and self-directed learning in the Other Learning Experiences.

However, significant curricular and evaluation system changes under the NSS curriculum have elicited uncertainties that often trigger stress in students. First, the total number of secondary education years was shortened, leaving students with less time to prepare for the public examination. Second, students may feel unfamiliar with the new examination requirements and, thus, feel less confident and find the need to seek private tutors’ help (Fung et al., 2017). Not only did students face challenges, studies found that teachers also possessed inadequate understanding of the reform and experienced difficulties in meeting students’ diverse learning needs (Cheung and Wong, 2012). Third, the introduction of “Liberal Studies” elicited a heated discussion on the gaps between the subject’s aspirations and actual implementation (Fok, 2016). Finally, as Chinese people strongly emphasize academic excellence, academic stress is a chronic challenge faced by Chinese adolescents (Chyu and Chen, 2022).

Hong Kong students have shown relatively lower levels of satisfaction and well-being compared with their counterparts in other countries (OECD, 2017). Under Hong Kong’s examination-oriented culture, students strive for academic excellence and experience heavy academic pressure to succeed. In addition, studies showed that despite having higher academic achievements, Hong Kong students had lower levels of self-efficacy (OECD, 2019). Although researchers and policymakers have examined students’ development under the NSS curriculum from the perspective of policy and reform evaluations, students’ perceptions and satisfaction with the NSS curriculum have received less attention, which suggests a strong need to examine Hong Kong students’ academic satisfaction with the NSS curriculum.

### Literature gaps and research questions

Although studies have examined student learning and development under the NSS curriculum, efforts to understand reform implementation have not yet revealed a holistic picture, and some research gaps remain to be addressed (Zhan et al., 2016).

The first research gap is that when understanding the NSS reform’s effectiveness, most studies mainly have adopted the reform executor perspective in the school settings (i.e., school heads and teachers), while ignoring students’ views of the NSS curriculum. For example, due to education reform’s top-down nature, extant research often focuses on identifying difficulties in the classroom; discrepancies between school leaders, teachers, and policymakers’ perceptions; and the characteristics of effective schooling (Cheng and Mok, 2008; Yuen et al., 2012; Cheung et al., 2019). However, secondary school students’ voices have largely been overlooked. Considering that students are the direct recipients of education services, their evaluations of curriculum and school programs reflect their academic satisfaction, which is an important indicator for measuring education reform’s success (Wach et al., 2016; Shek and Chai, 2020). Also, although this particular reform is expected to reduce academic stress compared with the former exam-oriented system, significant changes to curriculum and assessment schemes may pose additional stress for students (Shek and Chai, 2020). For example, Shek and Chai (2020) found that student’s stress levels ranged from moderate to severe. To sum up, because of the importance of students’ academic satisfaction and the prevalence of students’ academic stress, their relationship under the NSS curriculum needs to be examined.

The second research gap is that most studies have used a cross-sectional design, rendering them unable to measure environmental and individual factors’ longitudinal influence on students’ academic satisfaction. A longitudinal design helps in unraveling the effects of ecological factors on academic well-being. For example, by comparing its cross-sectional and longitudinal studies, Lent et al. (2012) stressed that the longitudinal study produced a more restricted set of relationships: Support, but not self-efficacy, at Time 1 predicted academic satisfaction at Time 2; while self-efficacy, but not support, at Time 1 predicted academic stress at Time 2. Moreover, although existing research has revealed that the NSS curriculum is a stressor for students and teachers (Cheung and Wong, 2012), students’ perceptions of and responses to stressful situations will change over time. On the one hand, students’ academic stress may gradually increase when they enter the senior secondary level (Liu and Lu, 2011). On the other hand, adolescents experience significant changes in their cognitive, emotional, and inter-personal development, and potentially acquire more protective resources at both the individual and environmental levels to respond to stress actively (Shek et al., 2022b). Nevertheless, longitudinal studies remain lacking on Hong Kong students’ academic satisfaction and its correlates under the NSS curriculum.

The above discussion indicates a need to investigate empirically how academic stress, school support, PYD attributes, and academic confidence shape students’ academic satisfaction based on students’ perspective. This study aims to address the following research questions by using a longitudinal study’s data on secondary school students:

Research Question 1: Is perceived academic stress related to students’ academic satisfaction? Based on previous research (Shek and Chai, 2020), we hypothesized that academic stress would be related to academic satisfaction negatively (Hypothesis 1).

Research Question 2: Is school support related to students’ academic satisfaction? Based on previous evidence (Torsheim and Wold, 2001), we hypothesized that school support would be related to academic satisfaction positively (Hypothesis 2).

Research Question 3: Are PYD attributes related to students’ academic satisfaction? Based on previous findings (Shek and Chai, 2020), we hypothesized that PYD attributes would be related to academic satisfaction positively (Hypothesis 3).

Research Question 4: Is academic confidence related to students’ academic satisfaction? Based on previous studies (Pajares and Schunk, 2001; DeWitz and Walsh, 2002), we hypothesized that academic confidence would be related to academic satisfaction positively (Hypothesis 4).

Research Question 5: Are academic stress, school support, and PYD attributes related to students’ confidence? Based on previous results (Burnett and Fanshawe, 1997; Sarid et al., 2004; Liu and Lu, 2011), we hypothesized a negative relationship between academic stress and students’ confidence (Hypothesis 5a), and positive relationships between school support and PYD attributes and students’ confidence (Hypotheses 5b and 5c, respectively).

**Figure 1:**
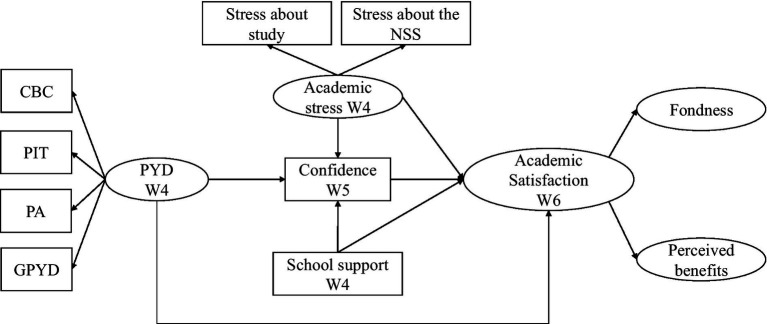
Hypothesized SEM model.

Research Question 6: Does academic confidence serve as a mediator in the links between academic stress, PYD, school support, and students’ academic satisfaction? Based on previous research (Pajares and Schunk, 2001; DeWitz and Walsh, 2002), we hypothesized that academic confidence mediated the relationships between academic stress, PYD attributes, school support, and students’ academic satisfaction (Hypotheses 6a–c, respectively). The hypothesized model is provided in Figure 1.

## Materials and methods

### Participants

This study used the data derived from a large-scale project initially launched in 2009–2010 to understand Hong Kong adolescents’ development. The original project collected data from Secondary 1 students in 28 secondary schools over 6 consecutive years, while only data from the three senior secondary school years were used in this study (i.e., Waves 4–6). Students responded to a questionnaire concerning different aspects of their development, including their perceptions of school support, PYD attributes, academic stress, academic confidence, and satisfaction with the NSS curriculum. This project utilized data collected from 2,023 students (*M*_age_ at Wave 1 = 12.53 years, *SD*_age_ = 0.66), including 959 boys (47.4%), 1,040 girls (51.4%), and 24 students (1.2%) who did not report their gender at Wave 1.

### Instruments

#### Academic stress

Two items were used to measure students’ perceived academic stress, including “Do you feel pressure in your current studies?” and “Do you feel pressure under the new senior secondary school curriculum?” (Shek and Chai, 2020). The first question assessed students’ subjective experience of stress in their academic studies in general (Putwain, 2007), and the second one evaluated students’ subjective experience of pressure with the NSS curriculum. The two items were rated on a four-point Likert scale (1 = “not at all” to 4 = “very much”). Higher ratings indicated higher levels of students’ perceived academic stress. This scale showed good psychometric properties (Shek and Chai, 2020). Data on academic stress collected at Wave 4 (i.e., Secondary 4) were used, indicating the initial level of perceived academic stress reported by students when entering senior secondary school education.

#### School support

A single item was developed to measure students’ perceived school support: “Does your school provide sufficient support for your studies?” A four-point Likert scale was used (1 = “quite insufficient” to 4 = “quite sufficient”). A higher rating suggested a higher level of perceived school support. Data collected at Wave 4 (i.e., Secondary 4) were used in this study.

#### Positive youth development attributes

Positive youth development attributes were evaluated using a shortened version of the Chinese Positive Youth Development Scale (CPYDS; Shek et al., 2007), comprising 44 items assessing 15 attributes of positive development based on review of Catalano et al. (2016): “bonding;” “resilience;” “social competence;” “recognition for positive behavior”; “emotional competence;” “cognitive competence;” “behavioral competence;” “moral competence;” “self-determination;” “self-efficacy;” “clear and positive identity;” “beliefs in the future;” “prosocial norms;” “prosocial involvement;” and “spirituality.” Shek and Ma (2010) further subsumed these 15 PYD attributes under four higher-order factors, which are cognitive-behavioral competencies (CBC), prosocial attributes (PA), positive identity (PIT), and general positive youth development qualities (GPYD). Some sample items of this scale included “when facing difficulties, I do not give up easily,” “I can get along with other people,” and “if I’m not happy, I can express my emotions properly.” The items in the “spirituality” subscale were rated on a seven-point scale (1 = “my life is very boring/empty” to 7 = “my life is full of energy/excitement”). All other subscales were rated on a six-point scale (1 = “strongly disagree” to 6 = “strongly agree”). Previous studies have indicated that this scale was reliable and valid (Shek et al., 2007; Shek and Ma, 2010). Data collected at Wave 4 (i.e., Secondary 4) were used for PYD attributes in this study.

#### Academic confidence

A single item was developed to evaluate students’ perceived confidence in meeting the NSS curriculum’s requirements, asking “are you confident in fulfilling the requirements of the NSS curriculum?” and using a four-point scale (1 = “no confidence” to 4 = “completely confident”). Higher scores indicated stronger confidence levels in handling the NSS curriculum. Data collected at Wave 5 (i.e., Secondary 5) were used for academic confidence.

#### Academic satisfaction

Students’ academic satisfaction was measured by evaluating students’ perceptions of the NSS curriculum. Altogether, 12 items were developed to measure students’ academic satisfaction level with the NSS curriculum on a six-point scale (1 = “strongly disagree” to 6 = “strongly agree”). After removing three reverse-coded items that indicated low item-total correlations from each subscale (Shek and Chai, 2020), the refined scale includes nine items under two factors: students’ fondness for and interest in the NSS curriculum (four items for the subscale, e.g., “I like the new senior secondary school curriculum”) and students’ perceptions of the benefits received from the NSS curriculum in promoting positive and holistic development (five items for the subscale, e.g., “the curriculum can help me establish positive values and attitudes”). Data at Wave 6 (i.e., Secondary 6) were used to estimate students’ academic satisfaction under the NSS curriculum. This scale showed good psychometric properties (Shek and Chai, 2020). The results of exploratory factor analysis, confirmatory factor analysis, and measurement invariance were reported in other publications (Shek and Chai, 2020; Dou and Shek, 2022).

### Data analysis plan

We conducted structural equation modeling (SEM) to test the hypothesized model *via* lavaan in R software. The model included two observed variables (i.e., school support and perceived confidence) and three latent variables (i.e., perceived academic stress, PYD attributes, and academic satisfaction). The indicators evaluating the model’s goodness of fit included comparative fit index (CFI), goodness-of-fit index (GFI), non-normed fit index (NNFI), and root mean square error of approximation (RMSEA). Following Kline’s suggestion (Kline, 2015), the adequate model fit values were above 0.90 for CFI, GFI, and NNFI, but below 0.08 for RMSEA. We conducted bootstrapping procedures on 5,000 resamples to calculate the effects at a 95% confidence interval (CI; Preacher and Hayes, 2008).

## Results

### Descriptive statistics

Table 1 summarizes the reliability, mean, standard deviation (SD), and correlations between the research variables. All scales used in the present study presented good reliability, with Cronbach’s alphas ranging from 0.89 to 0.96. In line with our hypotheses, academic stress was related negatively to confidence (*r* = −0.293, *p* < 0.001) and academic satisfaction (*r* = −0.176, *p* < 0.001). School support and PYD attributes were related positively to confidence (*r* = 0.154 and *r* = 0.366, respectively; *ps* < 0.001) and academic satisfaction (*r* = 0.209 and *r* = 0.318, *ps* < 0.001, respectively). Furthermore, confidence was related positively to academic satisfaction (*r* = 0.313, *p* < 0.001).

**Table 1:** Descriptive statistics and correlations.

		Cronbach’s alpha	Mean	SD	Correlations			
					1	2	3	4
1	Academic stress W4	0.888	2.767	0.717				
2	School support W4		2.581	0.646	−0.179^***^			
3	PYD attributes W4	0.960	4.479	0.594	−0.165^***^	0.175^***^		
4	Academic confidence W5		2.518	0.682	−0.293^***^	0.154^***^	0.366^***^	
5	Academic satisfaction W6	0.957	3.630	1.053	−0.176^***^	0.209^***^	0.318^***^	0.313^***^

^***^p < 0.001. W4 = Wave 4; W5 = Wave 5; W6 = Wave 6.

### Relationships between academic factors, PYD attributes, and students’ perceptions of the NSS curriculum

**Figure 2:**
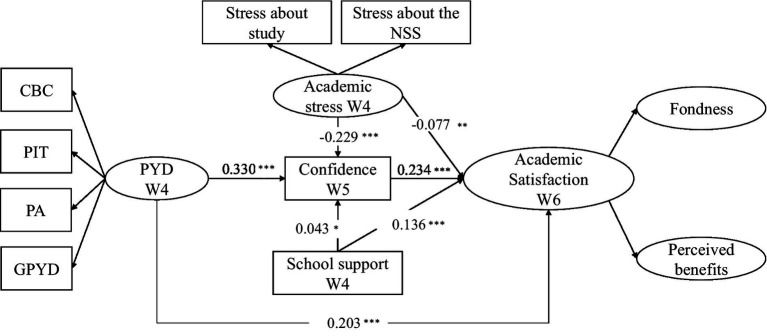
SEM model with standardized estimates. ^***^p < 0.001, ^**^p < 0.01, and ^*^p < 0.05.

The SEM model yielded a good fit (*χ^2^* = 817.75, *df* = 107, CFI = 0.973, GFI = 0.944, NNFI = 0.965, RMSEA = 0.060, 90% CI = [0.057, 0.064], AIC = 55181.091, SRMR = 0.051). As shown in Figure 2, academic stress at Wave 4 negatively predicted academic satisfaction at Wave 6 (direct effect = −0.077, *p* < 0.01; see Table 2). In line with our hypotheses, PYD attributes and school support at Wave 4 positively predicted academic satisfaction at Wave 6 (direct effect = 0.203 and 0.136, respectively; *p*s < 0.001). Furthermore, academic confidence at Wave 5 positively predicted academic satisfaction at Wave 6 (direct effect = 0.234, *p* < 0.001). The results also were consistent with the correlational findings (see Table 1). Thus, Hypotheses 1, 2, 3, and 4 were supported.

**Table 2:** Estimated predicting effects of academic factors, PYD attributes with the mediating effect of confidence in SEM.

Regression	B	SE	*β*	*p*
Academic stress ≥ Confidence	−0.244	0.024	−0.229	0.000
School support ≥ Confidence	0.045	0.023	0.043	0.043
PYD ≥ Confidence	0.433	0.030	0.330	0.000
Academic stress ≥ Academic satisfaction	−0.132	0.042	−0.077	0.002
Confidence ≥ Academic satisfaction	0.375	0.04	0.234	0.000
School support ≥ Academic satisfaction	0.231	0.038	0.136	0.000
PYD ≥ Academic satisfaction	0.428	0.054	0.203	0.000

### Relationships between academic stress, PYD attributes, school support, and academic confidence

As shown in Table 2, academic stress at Wave 4 negatively predicted academic confidence at Wave 5 (direct effect *β* = −0.229, *p* < 0.001). Moreover, PYD attributes and school support at Wave 4 positively predicted academic confidence at Wave 5 (direct effect *β* = 0.330, *p* < 0.001; direct effect *β* = 0.043, *p* < 0.05, respectively). Thus, Hypotheses 5a–c were supported.

### Academic confidence’s mediating role

We conducted bootstrapping procedures on 5,000 replicates to examine whether confidence mediated the relationships between academic stress, school support, PYD attributes, and academic satisfaction. As shown in Table 3, we found a significant indirect effect of academic stress on academic satisfaction *via* confidence (indirect effect = −0.092, *p* < 0.001). Furthermore, we also observed a significant indirect effect of PYD attributes on students’ academic satisfaction through confidence (indirect effect = 0.163, *p* < 0.001). Thus, the indirect effects of academic stress and PYD attributes on students’ academic satisfaction *via* academic confidence were proven robust. As the direct effects of academic stress and PYD attributes on students’ academic satisfaction remained significant, confidence partially mediated the negative relationship between academic stress and academic satisfaction, as well as the positive relationship between PYD attributes and academic satisfaction. However, academic confidence did not mediate the relationship between school support and academic satisfaction. As shown in Table 3, the lower and upper CI intervals contained zeroes, suggesting insufficient evidence of the mediating effect. In short, Hypotheses 6a and 6b were supported, but 6c was rejected.

**Table 3:** Results of bias-corrected bootstrap estimation for the mediation model.

Regression (on students’ academic satisfaction)	Point estimation	95% CI	
		Lower	Upper
Indirect effect of academic stress (confidence as mediator)	−0.092	−0.126	−0.06
Indirect effect of school support (confidence as mediator)	0.017	−0.002	0.038
Indirect effect of PYD attributes (confidence as mediator)	0.163	0.117	0.216
Direct effect of academic stress	−0.132	−0.226	−0.041
Direct effect of school support	0.231	0.143	0.318
Direct effect of PYD attributes	0.428	0.299	0.563
Total effect of academic stress	−0.223	−0.319	−0.128
Total effect of school support	0.248	0.158	0.337
Total effect of PYD attributes	0.591	0.464	0.722

## Discussion

It has been over a decade since the launch of education reform in Hong Kong in 2009. Research examining the reform’s implementation progress revealed obstacles and challenges, e.g., teachers’ heavy workloads and students’ diverse learning needs (Cheung and Wong, 2012). However, extant research concerning students’ perspectives on this reform remains scarce (Cheung et al., 2019).

For Research Questions 1–4 (i.e., ecological predictors of academic satisfaction), this study found that academic stress at Wave 4 negatively predicted academic satisfaction at Wave 6, while PYD attributes and school support at Wave 4 positively predicted academic satisfaction at Wave 6. Furthermore, academic confidence at Wave 5 positively predicted academic satisfaction at Wave 6. The findings echoed previous research results revealing negative relationship between perceived stress and students’ academic outcomes such as performance and satisfaction (Gavin et al., 2010; Ma, 2020). It is argued that students who experienced heavy stress tend to report lower learning motivation and less school engagement, which negatively linked to school satisfaction. Although some longitudinal studies found that a certain level of academic stress may enhance performance (Sarid et al., 2004), our findings found a negative relationship between academic stress with students’ satisfaction with the curriculum. Moreover, the present study’s findings also corresponded with theoretical arguments that academic confidence, school support, and positive attributes positively predict academic satisfaction. Sadeghi and Mahdavi (2020) found that self-efficacy, goal progress, environmental supports, and resources, directly and indirectly, were related to academic satisfaction. Environmental supports and resources refer to support that students receive in the academic and social domains, comparable with this study’s school support measure. Focusing on Bandura’s self-efficacy theory, DeWitz and Walsh (2002) found that college students’ self-efficacy beliefs were associated with their satisfaction with college life. The present study provided empirical evidence to fill the current literature gap in longitudinal research.

For Research Question 5, this study found a negative relationship between academic stress at Wave 4 and students’ confidence at Wave 5, and positive relationships between school support and PYD attributes at Wave 4 and students’ confidence at Wave 5, which echoed previous findings and theoretical arguments (e.g., Zorkina and Nalbone, 2003). For example, Shek and Wu (2016) found the prediction of positive youth development on academic confidence. Transactional model of stress and coping of Lazarus (1999) helps in understanding how the aforementioned ecological factors influence students’ academic confidence. The newly implemented curricula and assessment scheme under the NSS curriculum represent a source of considerable stress for Hong Kong students. Stress occurs when students perceive their coping resources, e.g., PYD attributes and school support, as inadequate. Students under heavy academic pressure, e.g., under education reform, often experience learning difficulties and experience less motivation and confidence, leading to diminished academic performance and lower satisfaction with school life (Burnett and Fanshawe, 1997; Sarid et al., 2004; Liu and Lu, 2011). When students experience stressful situations, if they seek or have sufficient social (e.g., school support) and psychological (e.g., resilience) resources, they may feel more confidence to cope with stress actively (Lazarus and Folkman, 1984).

For Research Question 6, this study found that academic confidence at Wave 5 mediated the relationships between academic stress and PYD attributes at Wave 4 and students’ academic satisfaction at Wave 6, which was in line with previous findings. For example, Garriott et al. (2015) proved that environmental supports predicted outcome expectations, self-efficacy, and academic satisfaction. In addition, outcome expectations predicted academic satisfaction. Similarly, longitudinal study of Xiang et al. (2019) also found the mediating role of self-esteem at Wave 2 in the effects of academic stress at Wave 1 on students’ psychological well-being at Wave 3. Our results corresponded with findings in existing literature and demonstrated academic confidence’s mediating role in the relationships between academic stress, PYD attributes, and students’ academic satisfaction.

Noteworthily, the present study found that academic confidence’s mediating role at Wave 5 in the relationships between school support at Wave 4 and students’ academic satisfaction at Wave 6 was insignificant. One possible explanation is that in the social context of Hong Kong, school support, particularly care from class teachers, may not be easily provided because the class size in Hong Kong is often large (with more than 30 students per class), and thus the workload of secondary school teachers is very heavy. This echoes the observation of Avidov-Ungar and Arviv-Elyashiv (2020) that teachers often took moderately negative or neutral stance toward education reform, unless they perceive higher levels of school readiness for reform and school support mechanisms. Other literature indicates a more complicated path from school support to academic satisfaction. For example, Danielsen et al. (2009) found that students’ scholastic competence mediated the relationship between social support from parents, teachers, and peers, and students’ life satisfaction. However, Han et al. (2021) found that self-efficacy only mediated the influence of student cohesiveness and student involvement, but not the influence of teacher support, on students’ satisfaction with learning. These findings revealed the potentially more complicated relationship between school support, students’ academic confidence, and academic satisfaction, which calls for further studies.

The present study has several theoretical implications. The findings clarified the relationship between academic stress and academic satisfaction by using longitudinal data collected over the whole secondary school education period. This is the first study that investigated Hong Kong students’ academic stress and satisfaction over time. Only study of Leung et al. (2021) examined how academic achievement and positive social relationships predicted primary students’ well-being by using a longitudinal survey and collecting data at two time points within a year. Furthermore, our findings provide empirical evidence for clarifying conceptual pathways through which school and individual factors shape students’ academic satisfaction, which sheds light on promoting students’ well-being in examination-oriented cultures, particularly in Chinese societies. Reducing academic stress and enhancing academic satisfaction are essential to students’ academic well-being. As for longitudinal studies, only Shek and Chai (2020) examined the relationship between students’ PYD qualities, life satisfaction, academic stress, and academic satisfaction. Few studies have analyzed students’ academic satisfaction and its related school and individual factors comprehensively. This study offers empirical evidence to clarify the related conceptual pathways. In view of the paucity of related studies in different Chinese contexts (Shek et al., 2022a), this is an important study to the limited Chinese scientific literature.

This study also carries practical implications for promoting students’ academic well-being. By identifying critical school and individual resources that influence academic satisfaction, school administrators and social workers can better provide support to build students’ confidence and subsequently enhance their academic well-being. Schools are required to incorporate the teaching of generic skills and positive values and attitudes into the subject curriculum. The guidance work in schools also emphasizes helping students understand their abilities and emotions better, as well as enhancing their self-confidence and self-esteem (Education Bureau, 2015). Many of the personal qualities emphasized in these programs overlap with PYD attributes, but scholars have identified several problems in the school organizational structure or in the curriculum design when implementing these curricular components (Ha et al., 2008; Lam and Hui, 2010; Cheung and Wong, 2012; Leung et al., 2014; Luk and Lin, 2015). Therefore, the present study could provide empirical evidence to improve these programs in the future. Furthermore, the findings shed light on the refinement of school-based positive youth development programs. In particular, the Project P.A.T.H.S. program was a school-based positive youth development program implemented in 2005. The evaluation’s findings indicated that the program has promoted Hong Kong adolescents’ positive development and reduced their risk behavior (Shek and Wu, 2016). The present study could offer insights to develop similar programs in the future. However, although different stakeholders regarded PYD atttibutes to be important, systematic effort to promote PYD attributes in adolescents in Hong Kong is grossly inadequate (Shek et al., 2021).

This project also carries policy implications. As revealed in earlier research, significant discrepancies were found between the perceptions of education reform of different stakeholders (Yuen et al., 2012; Cheung et al., 2019). Understanding students’ perceptions of the education reform measures and their well-being can provide a knowledge base for policy recommendations. Furthermore, local policy documents did not emphasize the “student well-being” concept. Some policy documents focused on students’ overall study hours and sleeping time (Chu, 2018), and did not fully address students’ schoolwork-related stress and satisfaction. This demonstrated room for improvement in policy support on students’ well-being in Hong Kong. Our findings illustrated the relationship between students’ academic stress and their academic satisfaction, which can support the planning for future policies.

Despite the theoretical, practical, and policy implication, this study has some limitations. First, aside from stress, school support, PYD attributes, and confidence, other psychological processes may form different paths that affect students’ academic satisfaction. Specific aspects of the PYD attributes may exert a stronger influence on students’ academic satisfaction. Factors such as student cohesiveness and student involvement also remain under-examined. Second, we only collected students’ self-reported data in this study. Although self-reported data can reflect students’ perspectives, methodological bias might be present. For PYD attributes’ measures, data reported by students’ parents, teachers, and peers would help address such methodological bias. For school support measures, objective assessment of the academic support that teachers provide can complement students’ self-reported data. Third, two single-item scales, including school support and academic confidence, were used in the present study. Although researchers argue that a well-designed single-item scale can be sufficient (Bergkvist and Rossiter, 2007), there are concerns about whether a single item can fully represent a complex concept with multiple dimensions. Future studies may consider using multiple-item scales to capture different facets of these factors. Finally, the data were collected within a non-pandemic context. As COVID-19 has led to school lockdown and forced reliance on online teaching and learning, such changes have altered the learning context of young people and would lead to many issues (Shek, 2021). Hence, effort to replicate the present study in a COVID-19 context would be exciting.

## Conclusion

The present study generally provides support for previous findings on the relationships between academic stress, school support, PYD attributes, academic confidence, and academic satisfaction. Our findings emphasize the prominence of PYD attributes, school support, and confidence in enhancing students’ academic satisfaction. We suggested that strengthening students’ PYD attributes and confidence, as well as increasing school support, can improve students’ healthy development and academic well-being. This study fills the literature gap of the lack of longitudinal empirical research on academic well-being, and it provides evidence to support the development of students’ positive attributes and confidence in senior secondary schools. Practitioners and policymakers also can benefit from utilizing the findings to improve the NSS curriculum and guidance work in schools, or even develop positive school-based youth development programs.

## Data availability statement

The raw data supporting the conclusions of this article will be made available by the authors, without undue reservation.

## Ethics statement

The studies involving human participants were reviewed and approved by the Human Subjects Ethics Sub-committee of the Hong Kong Polytechnic University. Written informed consent to participate in this study was provided by the participants’ legal guardian/next of kin.

## Author contributions

DD and DS: conceptualization, methodology, project administration, and funding acquisition. DD: formal analysis. DD, TW, and DS: writing–original draft preparation and writing-review and editing. DS: supervision. All authors contributed to the article and approved the submitted version.

## Funding

The Project P.A.T.H.S. was financially supported by the Hong Kong Jockey Club Charities Trust. This paper was financially supported by Wofoo Foundation, Research Matching Fund of the Research Grants Council (1.54.xx.52YW), and the start-up grant of the DD (Project ID: P0035101).

## Conflict of interest

The authors declare that the research was conducted in the absence of any commercial or financial relationships that could be construed as a potential conflict of interest.

## Publisher’s note

All claims expressed in this article are solely those of the authors and do not necessarily represent those of their affiliated organizations, or those of the publisher, the editors and the reviewers. Any product that may be evaluated in this article, or claim that may be made by its manufacturer, is not guaranteed or endorsed by the publisher.

## References

[ref1] Archambault I. Janosz M. Morizot J. Pagani L. (2009). Adolescent behavioral, affective and cognitive engagement in schools: relationships to dropout. J. Sch. Health 79, 408–415. doi: 10.1111/j.1746-1561.2009.00428.x, PMID: 19691715

[ref2] Avidov-Ungar O. Arviv-Elyashiv R. (2020). Teachers’ perceptions of educational reform: the schools’ readiness, supporting mechanisms and contributions of the reform. Int. J. Educ. Manag. 35, 173–187. doi: 10.1108/IJEM-12-2018-0386

[ref3] Bandura A. (1977). Self-efficacy: toward a unifying theory of behavioral change. Psychol. Rev. 84, 191–215. doi: 10.1037/0033-295X.84.2.191, PMID: 847061

[ref4] Benson P. L. Scales P. C. Hamilton S. F. Sesma A. (2007). “Positive youth development: theory, research, and applications,” in Handbook of Child Psychology (Volume 1: Theoretical Models of Human Development), 6th edn. eds. Damon W. Lerner R. M. Lerner R. M. (Hoboken, N.J.: John Wiley & Sons. Inc.), 894–941.

[ref5] Benson P. L. Scales P. C. Syvertsen A. K. (2011). Chapter 8- the contribution of the developmental assets framework to positive youth development theory and practice. Adv. Child Dev. Behav. 41, 197–230. doi: 10.1016/B978-0-12-386492-5.00008-7 23259193

[ref6] Bergkvist L. Rossiter J. R. (2007). The predictive validity of multiple-item versus single-item measures of the same constructs. J. Mark. Res. 44, 175–184. doi: 10.1509/jmkr.44.2.175

[ref7] Bronfenbrenner U. (1979). Contexts of child rearing: problems and prospects. Am. Psychol. 34, 844–850. doi: 10.1037/0003-066X.34.10.844

[ref8] Burnett P. C. Fanshawe J. P. (1997). Measuring school-related stressors in adoelscents. J. Youth Adolesc. 26, 415–428. doi: 10.1023/A:1024529321194

[ref9] Byrne M. Flood B. (2003). Assessing the teaching quality of accounting programmes: an evaluation of the course experience questionnaire. Assess. Eval. High. Educ. 28, 135–145. doi: 10.1080/02602930301668

[ref10] Catalano R. F. Berglund M. L. Ryan J. A. M. Lonczak H. S. Hawkins J. D. (2016). Positive youth development in the United States: research findings on evaluations of positive youth development programs. Ann. Am. Acad. Pol. Soc. Sci. 591, 98–124. doi: 10.1177/0002716203260102

[ref11] Chapin L. A. (2015). Mexican-American boys' positive outcomes and resilience: importance of social support and individual attributes. J. Child Fam. Stud. 24, 1791–1799. doi: 10.1007/s10826-014-9982-8

[ref12] Chemers M. M. Hu L. T. Garcia B. F. (2001). Academic self-efficacy and first year college student performance and adjustment. J. Educ. Psychol. 93, 55–64. doi: 10.1037/0022-0663.93.1.55

[ref13] Cheng Y. C. Mok M. M. C. (2008). What effective classroom? Towards a paradigm shift. Sch. Eff. Sch. Improv. 19, 365–385. doi: 10.1080/09243450802535174

[ref14] Cheung A. C. K. Keung C. P. C. Mak B. S. Y. (2019). Examining the key stakeholders' perception of student learning: towards a paradigm shift in secondary education in Hong Kong. Asia Pac. J. Educ. 39, 532–547. doi: 10.1080/02188791.2019.1604318

[ref15] Cheung A. C. K. Wong P. M. (2012). Factors affecting the implementation of curriculum reform in Hong Kong: key findings from a large-scale survey study. Int. J. Educ. Manag. 26, 39–54. doi: 10.1108/09513541211194374

[ref16] Chu K. (2018). Overall study hours and student well-being in Hong Kong (IN05/17-18). Research Office, Legislative Council Secretariat. Available at: https://www.legco.gov.hk/research-publications/english/1718in05-overall-study-hours-and-student-well-being-in-hong-kong-20180130-e.pdf (Accessed March 9, 2022).

[ref17] Chyu E. P. Y. Chen J. K. (2022). The correlates of academic stress in Hong Kong. Int. J. Environ. Res. Public Health 19:4009. doi: 10.3390/ijerph19074009, PMID: 35409692 PMC8997729

[ref18] Currie C. Zanotti C. Morgan A. Currie D. de Looze M. Roberts C.. (eds.) (2012). Social determinants of health and well-being among young people. Health behaviour in school-aged children (HBSC) study: International report from the 2009/2010 survey. WHO regional Office for Europe. Available at: http://www.euro.who.int/__data/assets/pdf_file/0003/163857/Social-determinants-of-health-and-well-being-among-young-people.pdf (Accessed March 9, 2022).

[ref19] Damon W. (2016). What is positive youth development? Ann. Am. Acad. Pol. Soc. Sci. 591, 13–24. doi: 10.1177/0002716203260092

[ref20] Danielsen A. G. Samdal O. Hetland J. Wold B. (2009). School-related social support and students' perceived life satisfaction. J. Educ. Res. 102, 303–320. doi: 10.3200/joer.102.4.303-320

[ref21] Deci E. L. Ryan R. M. (2008). Self-determination theory: a macrotheory of human motivation, development, and health. Can. Psychol. 49, 182–185. doi: 10.1037/a0012801

[ref22] DeWitz S. J. Walsh W. B. (2002). Self-efficacy and college student satisfaction. J. Career Assess. 10, 315–326. doi: 10.1177/10672702010003003

[ref23] Dou D. Shek D. T. L. (2022). Hong Kong high school students’ perceptions of the new secondary school curriculum. Front. Pediatr. 10:881515. doi: 10.3389/fped.2022.881515, PMID: 35935353 PMC9354656

[ref24] Education Bureau (2015). Student guidance in secondary schools. Education Bureau. Available at: https://www.edb.gov.hk/en/teacher/student-guidance-discipline-services/projects-services/sgs/guidance-in-secondary-schools/index.html (Accessed March 9, 2022).

[ref25] Fariborz N. Hadi J. Taghvaie N. A. (2019). Students' academic stress, stress response and academic burnout: mediating role of self-efficacy. Pertan. J. Soc. Sci. Hum. 27, 2441–2454.

[ref26] Flores A. (2007). Attributional style, self-efficacy, and stress as predictors of academic success and academic satisfaction in college students. Dissertation/Ph.D. thesis. Salt Lake City(UT): The University of Utah.

[ref27] Fok P. K. (2016). Liberal studies reform in Hong Kong secondary education: contrasting desirability with feasibility. Educ. Res. Policy Prac. 15, 209–230. doi: 10.1007/s10671-015-9190-3

[ref28] Fung D. Lui W. M. Liang T. Su A. (2017). The way forward for the development of liberal studies: how teachers perceive its introduction and implementation in Hong Kong secondary schools. Asia Pac. Educ. Rev. 18, 123–134. doi: 10.1007/s12564-017-9471-z

[ref29] Fung C. L. Yip W. Y. (2010). The policies of reintroducing liberal studies into Hong Kong secondary schools. Educ. Res. Policy Prac. 9, 17–40. doi: 10.1007/s10671-009-9076-3

[ref30] Garriott P. O. Hudyma A. Keene C. Santiago D. (2015). Social cognitive predictors of first-and non-first-generation college students' academic and life satisfaction. J. Couns. Psychol. 62, 253–263. doi: 10.1037/cou0000066, PMID: 25730170

[ref31] Gavin L. E. Catalano R. F. David-Ferdon C. Gloppen K. M. Markham C. M. (2010). A review of positive youth development programs that promote adolescent sexual and reproductive health. J. Adolesc. Health 46, S75–S91. doi: 10.1016/j.jadohealth.2009.11.215, PMID: 20172462

[ref32] Ha A. S. Wong A. C. Sum R. K. Chan D. W. (2008). Understanding teachers’ will and capacity to accomplish physical education curriculum reform: the implications for teacher development. Sport Educ. Soc. 13, 77–95. doi: 10.1080/13573320701780746

[ref33] Han J. Geng X. Wang Q. (2021). Sustainable development of university EFL learners’ engagement, satisfaction, and self-efficacy in online learning environments: Chinese experiences. Sustain. For. 13:11655. doi: 10.3390/su132111655

[ref34] Hui S. K. F. Cheung H. Y. Kennedy K. J. (2020). A critical review of the development of generic learning outcomes: how engaging is the new senior secondary (NSS) curriculum reform in Hong Kong? J. Educ. Hum. Dev. 9, 67–84. doi: 10.15640/jehd.v9n4a8

[ref35] Jadidian A. Duffy R. D. (2011). Work volition, career decision self-efficacy, and academic satisfaction. J. Career Assess. 20, 154–165. doi: 10.1177/1069072711420851

[ref36] Kline R. B. (2015). Principles and Practice of Structural Equation Modeling 4th Edn. New York: The Guilford Press

[ref37] Lam S. K. Y. Hui E. K. P. (2010). Factors affecting the involvement of teachers in guidance and counselling as a whole-school approach. Br. J. Guid. Couns. 38, 219–234. doi: 10.1080/03069881003674962

[ref38] Lazarus R. S. (1999). Stress and Emotion: A New Synthesis. New York: Springer Pub. Co

[ref39] Lazarus R. S. Folkman S. (1984). Stress, Appraisal, and Coping. New York: Springer Pub. Co

[ref40] Lent R. W. do Céu Taveira M. Figuera P. Dorio I. Faria S. Gonçalves A. M. (2017). Test of the social cognitive model of well-being in Spanish college students. J. Career Assess. 25, 135–143. doi: 10.1177/1069072716657821

[ref41] Lent R. W. do Céu Taveira M. Lobo C. (2012). Two tests of the social cognitive model of well-being in Portuguese college students. J. Vocat. Behav. 80, 362–371. doi: 10.1016/j.jvb.2011.08.009

[ref42] Lent R. W. Singley D. Sheu H.-B. Schmidt J. A. Schmidt L. C. (2016). Relation of social-cognitive factors to academic satisfaction in engineering students. J. Career Assess. 15, 87–97. doi: 10.1177/1069072706294518

[ref43] Lerner R. M. Lerner J. V. Almerigi J. B. Theokas C. Phelps E. Gestsdottir S.. (2005). Positive youth development, participation in community youth development programs, and community contributions of fifth-grade adolescents: findings from the first wave of the 4-H study of positive youth development. J. Early Adolesc. 25, 17–71. doi: 10.1177/0272431604272461

[ref44] Leung C. Leung J. T. Y. Kwok S. Y. C. L. Hui A. Lo H. Tam H. L.. (2021). Predictors to happiness in primary students: positive relationships or academic achievement. Appl. Res. Qual. Life 16, 2335–2349. doi: 10.1007/s11482-021-09928-4

[ref45] Leung K. C. Leung F. K. S. Zuo H. (2014). A study of the alignment of learning targets and assessment to generic skills in the new senior secondary mathematics curriculum in Hong Kong. Stud. Educ. Eval. 43, 115–132. doi: 10.1016/j.stueduc.2014.09.002

[ref46] Liu Y. Lu Z. (2011). The Chinese high school student’s stress in the school and academic achievement. Educ. Psychol. 31, 27–35. doi: 10.1080/01443410.2010.513959

[ref47] Lodi E. Boerchi D. Magnano P. Patrizi P. (2017). College satisfaction scale (CSS): evaluation of contextual satisfaction in relation to college student life satisfaction and academic performance. Appl. Psychol. Bull. 65, 51–64.

[ref48] Luk J. Lin A. (2015). Voices without words: doing critical literate talk in English as a second language. TESOL Q. 49, 67–91. doi: 10.1002/tesq.161

[ref49] Ma C. M. S. (2020). The relationship between social support and life satisfaction among Chinese and ethnic minority adolescents in Hong Kong: the mediating role of positive youth development. Child Indic. Res. 13, 659–679. doi: 10.1007/s12187-019-09638-2

[ref50] Mampane M. R. (2014). Factors contributing to the resilience of middle-adolescents in a south African township: insights from a resilience questionnaire. S. Afr. J. Educ. 34, 1–11. doi: 10.15700/201412052114

[ref51] Marchiondo K. Marchiondo L. A. Lasiter S. (2010). Faculty incivility: effects on program satisfaction of BSN students. J. Nurs. Educ. 49, 608–614. doi: 10.3928/01484834-20100524-05, PMID: 20509588

[ref52] Montero R. Gempp R. Vargas M. (2022). Chilean university students’ satisfaction with online learning during COVID-19 pandemic: demonstrating the two-layer methodology. Front. Psychol. 13:887891. doi: 10.3389/fpsyg.2022.887891, PMID: 35967724 PMC9364960

[ref53] Nauta M. M. (2007). Assessing college students’ satisfaction with their academic majors. J. Career Assess. 15, 446–462. doi: 10.1177/1069072707305762

[ref54] OECD (2017). PISA 2015 Result (Volume III): Students' Well-Being. Paris: PISA, OECD Publishing

[ref55] OECD (2019). PISA 2018 Results (Volume III): What School Life Means for Students’ Lives. Paris: PISA, OECD Publishing.

[ref56] Pajares F. (2007). “Motivational role of self-efficacy beliefs in self-regulated learning,” in Motivation and Self-Regulated Learning: Theory, Research, and Application. eds. Schunk D. H. Zimmerman B. J. (New York: Routledge), 111–139.

[ref57] Pajares F. Schunk D. (2001). “The development of academic self-efficacy,” in Development of Achievement Motivation. eds. Wigfield A. Eccles J. (San Diego: Academic Press), 1–27.

[ref58] Plenty S. Ostberg V. Almquist Y. B. Augustine L. Modin B. (2014). Psychosocial working conditions: an analysis of emotional symptoms and conduct problems amongst adolescent students. J. Adolesc. 37, 407–417. doi: 10.1016/j.adolescence.2014.03.008, PMID: 24793388

[ref59] Polat H. Karabatak S. (2022). Effect of flipped classroom model on academic achievement, academic satisfaction and general belongingness. Learn. Environ. Res. 25, 159–182. doi: 10.1007/s10984-021-09355-0

[ref60] Preacher K. J. Hayes A. F. (2008). Asymptotic and resampling strategies for assessing and comparing indirect effects in multiple mediator models. Behav. Res. Methods 40, 879–891. doi: 10.3758/brm.40.3.879, PMID: 18697684

[ref61] Putwain D. (2007). Researching academic stress and anxiety in students: some methodological considerations. Br. Educ. Res. J. 33, 207–219. doi: 10.1080/01411920701208258

[ref62] Rowe F. Stewart D. Stewart D. Patterson C. (2007). Promoting school connectedness through whole school approaches. Health Educ. 107, 524–542. doi: 10.1108/09654280710827920

[ref63] Ryan A. M. (2001). The peer group as a context for the development of young adolescent motivation and achievement. Child Dev. 72, 1135–1150. doi: 10.1111/1467-8624.00338, PMID: 11480938

[ref64] Sadeghi A. Mahdavi F. (2020). Social-cognitive predictors of Iranian college students’ academic well-being. J. Career Dev. 47, 579–591. doi: 10.1177/0894845319826275

[ref001] Sanders L. Burton J. D. (1996). From retention to satisfaction: New outcomes for assessing the freshman experience. Research in Higher Education 37, 555–567. doi: 10.1007/BF01724938

[ref65] Sander P. Sanders L. (2003). Measuring confidence in academic study: a summary report. Elect. J. Res. Educ. Psychol. Psychopedag. 1, 1–17.

[ref66] Sarid O. Anson O. Yaari A. Margalith M. (2004). Academic stress, immunological reaction, and academic performance among students of nursing and physiotherapy. Res. Nurs. Health 27, 370–377. doi: 10.1002/nur.20028, PMID: 15362147

[ref67] Shek D. T. L. (2021). COVID-19 and quality of life: twelve reflections. Appl. Res. Qual. Life 16, 1–11. doi: 10.1007/s11482-020-09898-z, PMID: 33425064 PMC7786317

[ref68] Shek D. T. L. Chai W. (2020). The impact of positive youth development attributes and life satisfaction on academic well-being: a longitudinal mediation study. Front. Psychol. 11:2126. doi: 10.3389/fpsyg.2020.02126, PMID: 32982869 PMC7490328

[ref69] Shek D. T. L. Dou D. Cheng M. N. S. (2022b). “Transition from adolescence to emerging adulthood,” in The Encyclopedia of Child and Adolescent Development (Volume 7: History, Theory, and Culture in adolescence). eds. Hupp S. Jewell J. (Hoboken, N.J.: John Wiley & Sons, Inc.), 3145–3154.

[ref70] Shek D. T. L. Dou D. Zhu X. Chai W. (2019). Positive youth development: current perspectives. Adolesc. Health Med. Ther. 10, 131–141. doi: 10.2147/AHMT.S179946, PMID: 31572041 PMC6756153

[ref71] Shek D. T. L. Lin L. Ma C. M. S. Yu L. Leung J. T. Y. Wu F. K. Y.. (2021). Perceptions of adolescents, teachers and parents of life skills education and life skills in high school students in Hong Kong. Appl. Res. Qual. Life 16, 1847–1860. doi: 10.1007/s11482-020-09848-9

[ref72] Shek D. T. L. Ma C. M. S. (2010). Dimensionality of the Chinese positive youth development scale: confirmatory factor analyses. Soc. Indic. Res. 98, 41–59. doi: 10.1007/s11205-009-9515-9

[ref73] Shek D. T. L. Peng H. Zhou Z. (2022a). Children and adolescent quality of life under socialism with Chinese characteristics. Appl. Res. Qual. Life 17, 2447–2453. doi: 10.1007/s11482-021-09999-3, PMID: 34567280 PMC8455227

[ref74] Shek D. T. L. Siu A. M. H. Lee T. Y. (2007). The Chinese positive youth development scale. Res. Soc. Work. Pract. 17, 380–391. doi: 10.1177/1049731506296196

[ref75] Shek D. T. L. Wu F. K. (2016). Positive youth development and academic behavior in Chinese secondary school students in Hong Kong. Int. J. Disab. Hum. Dev. 15, 455–459. doi: 10.1515/ijdhd-2017-5012

[ref76] Shochet I. M. Dadds M. R. Ham D. Montague R. (2006). School connectedness is an underemphasized parameter in adolescent mental health: results of a community prediction study. J. Clin. Child Adolesc. Psychol. 35, 170–179. doi: 10.1207/s15374424jccp3502_1, PMID: 16597213

[ref77] Simonsen I. E. Rundmo T. (2020). The role of school identification and self-efficacy in school satisfaction among Norwegian high-school students. Soc. Psychol. Educ. 23, 1565–1586. doi: 10.1007/s11218-020-09595-7

[ref78] Singh K. Granville M. Dika S. (2002). Mathematics and science achievement: effects of motivation, interest, and academic engagement. J. Educ. Res. 95, 323–332. doi: 10.1080/00220670209596607

[ref79] Suldo S. M. Shaffer E. J. Riley K. N. (2008). A social-cognitive-behavioral model of academic predictors of adolescents' life satisfaction. Sch. Psychol. Q. 23, 56–69. doi: 10.1037/1045-3830.23.1.56

[ref80] Torsheim T. Wold B. (2001). School-related stress, support, and subjective health complaints among early adolescents: a multilevel approach. J. Adolesc. 24, 701–713. doi: 10.1006/jado.2001.0440, PMID: 11790051

[ref81] Usher E. L. Pajares F. (2008). Self-efficacy for self-regulated learning: a validation study. Educ. Psychol. Meas. 68, 443–463. doi: 10.1177/0013164407308475

[ref82] Wach F. S. Karbach J. Ruffing S. Brunken R. Spinath F. M. (2016). University students' satisfaction with their academic studies: personality and motivation matter. Front. Psychol. 7:55. doi: 10.3389/fpsyg.2016.00055, PMID: 26909049 PMC4754397

[ref83] Wong T. K. Y. Siu A. F. Y. (2017). Relationships between school climate dimensions and adolescents’ school life satisfaction, academic satisfaction and perceived popularity within a Chinese context. Sch. Ment. Heal. 9, 237–248. doi: 10.1007/s12310-017-9209-4

[ref84] Xiang Z. Tan S. Kang Q. Zhang B. Zhu L. (2019). Longitudinal effects of examination stress on psychological well-being and a possible mediating role of self-esteem in Chinese high school students. J. Happiness Stud. 20, 283–305. doi: 10.1007/s10902-017-9948-9

[ref85] Yuen T. W. W. Cheung A. C. K. Wong P. M. (2012). A study of the impact of the first phase of the curriculum reform on student learning in Hong Kong. Int. J. Educ. Manag. 26, 710–728. doi: 10.1108/09513541211263782

[ref86] Zhan Y. So W. M. W. Cheng N. Y. I. (2016). Implementation matters: teachers’ pedagogical practices during the implementation of an interdisciplinary curriculum in Hong Kong. Asia Pac. Educ. Res. 25, 527–539. doi: 10.1007/s40299-016-0278-1

[ref87] Zorkina Y. Nalbone D. P. (2003). Effect of induced level of confidence on college students' performance on a cognitive test. Curr. Res. Soc. Psychol. 8, 148–162.

